# Work Adaptations Insufficient to Address Growing Heat Risk for U.S. Agricultural Workers

**DOI:** 10.1088/1748-9326/ab86f4

**Published:** 2020-08-25

**Authors:** Michelle Tigchelaar, David S. Battisti, June T. Spector

**Affiliations:** 1 Department of Atmospheric Sciences, University of Washington, Seattle, WA; 2 now at: Center for Ocean Solutions, Stanford University, Palo Alto, CA; 3 Department of Environmental and Occupational Health Sciences, University of Washington, Seattle, WA; 4 Department of Medicine, University of Washington, Seattle, WA

## Abstract

The over one million agricultural workers in the United States (U.S.) are amongst the populations most vulnerable to the health impacts of extreme heat. Climate change will further increase this vulnerability. Here we estimate the magnitude and spatial patterns of the growing heat exposure and health risk faced by U.S. crop workers and assess the effect of workplace adaptations on mitigating that risk. We find that the average number of days spent working in unsafe conditions will double by mid-century, and, without mitigation, triple by the end of it. Increases in rest time and the availability of climate-controlled recovery areas can eliminate this risk but could affect farm productivity, farm worker earnings, and/or labor costs much more than alternative measures. Safeguarding the health and well-being of U.S. crop workers will therefore require systemic change beyond the worker and workplace level.

## Introduction

The $45 billion worth of fruits, nuts, and vegetables produced annually in the United States (U.S.) (1) are planted, harvested, and processed by laborers at high risk of adverse health effects from heat exposure. In fact, U.S. crop workers are twenty times more likely to die from illnesses related to heat stress than U.S. civilian workers overall (2). Their elevated risk derives both from the nature of the work – outdoors and with high physical demands – and from compounding vulnerabilities such as poverty, migrant status, language barriers, and barriers to acceptable health care (3-5). Climate change will further increase the exposure of outdoor workers to extreme heat (6,7). A better understanding of the magnitude and spatial patterns of this growing heat exposure and health risk is necessary to guide adaptation planning (8).

Working in high heat poses a health risk because heat stress is an established cause of heat-related illnesses, including heat rash, heat cramps, heat syncope (fainting), and heat exhaustion (9). When human thermoregulatory responses are overwhelmed, severe heat-related illness and death from exertional heat stroke can occur (10). In addition to heat-related illness, occupational heat stress has been linked with increased risk for traumatic injuries (11) and acute kidney injury (12). Heat events have also been linked to adverse mental health outcomes (13). Already in present-day climate, reports from California, Florida, North Carolina, Oregon, and Washington suggest an increased risk of dehydration, kidney injury, and symptoms of heat-related illness among crop workers (12,14-17).

Heat stress is ultimately a function of the net exposure of workers to heat, which includes the ambient environment (air temperature, humidity, solar radiation, and wind speed), clothing, and the metabolic heat generated by physical activity. On an individual level, factors like age, chronic diseases, use of some medications, and certain beliefs about the treatment and prevention of heat-related illness may increase risk (9,18). Modifiable workplace factors generate additional risk for agricultural workers; for example, the absence of shade, limited opportunities to adequately hydrate, and payment structures such as piece-rate payment, which incentivizes working harder and minimizing breaks (19). Agricultural workers may be subject to hazardous working conditions, harmful living conditions, non-livable wages, and unfair labor management, with power structures and other structural vulnerabilities preventing workers from exerting control over workplace safety and health practices (20-22).

In the U.S., it is estimated there are over a million distinct hired crop workers, not counting the self-employed and unpaid (family) work (23,24). More than three-quarters of hired agricultural workers are foreign-born, predominantly in Mexico, and only about half of the workers are legally authorized to work in the U.S. (5). For those on work visas, the employer typically controls housing and travel arrangements (25). The average education level amongst U.S. crop workers is eighth grade, and 71% report not speaking English well. Fewer than half of workers have health insurance, and the cost of health care is the most-cited barrier to accessing health care: a third of farmworkers have family incomes below the federal poverty line (5).

Whereas studies of present-day heat exposure and response – e.g. in the U.S. (12,14-17), India (26), Central America (27,28), and Africa (29,30) – have largely been conducted at the local level, future projections have been mostly global or regional in scope (31-34). However, as highlighted above, the context-dependence of vulnerability and exposure calls for the development of more granular projections at policy-relevant scales (35,36). Furthermore, most projections have used declining labor productivity as the impact metric, but farm-level studies in the U.S. have found that crop workers often do not have control over work organization and are more likely to risk their health than to reduce their work effort (37,38), suggesting that in certain geographies heat risk is a more appropriate metric. In this study we focus on the U.S. and calculate increases in heat health risk for agricultural workers with 2 and 4 °C of global warming. We then, for the first time, quantify the effect of various adaptations at the workplace level on mitigating this increased risk.

## Methods

### Agricultural workers & vulnerability

County-level employment data were obtained from the Bureau of Labor Statistics (BLS) Quarterly Census of Employment and Wages (QCEW), which provides monthly employment levels in the categories defined by the North American Industry Classification System (NAICS) (24). For the purposes of this study, we used NAICS codes 111 (Crop production) and 1151 (Support activities for crop production). For each county, we averaged monthly employment levels from 2009-2018 and calculated the maximum number of workers over the May through September (MJJAS) growing season.

The BLS estimates that the QCEW represents only half of all agricultural workers (39). The QCEW employment data are derived from tax reports of employers subject to State unemployment insurance. It therefore excludes proprietors, the unincorporated self-employed, unpaid family members, and farm workers not covered by State insurance law, including undocumented workers (39,40). In 2017 for example, the QCEW recorded 1.3 million wage and salary agricultural workers, but did not cover an additional 0.4 million waged workers and 0.8 million self-employed workers (23,39). Other national surveys, such as the U.S. Department of Agriculture Census, have similar methodological shortcomings. As such, our study underestimates the absolute number of exposed workers. However, the QCEW data remain the best available employment numbers at the county level.

Most of the demographic characteristics of crop workers associated with elevated heat vulnerability are included in the Social Vulnerability Index developed by the U.S. Centers for Disease Control and Prevention to identify high-priority areas for improving disaster preparedness and response, including for climate-related hazards (41). We described the spatial distribution of U.S. agricultural workers using QCEW data and overlaid the Social Vulnerability Index at the county level.

### Climate data

‘Heat’ can be measured in multiple ways. For the purposes of health impacts in working populations, the Wet Bulb Globe Temperature (WBGT) is considered the gold standard, used by government agencies and professional organizations including the National Institute for Occupational Safety and Health (NIOSH) (42) and the American Conference of Governmental Industrial Hygienists (ACGIH) (43). However, WBGT is difficult to measure, difficult to estimate from climate projections (44), and difficult to incorporate into risk communication. The Heat Index (HI), which is a function of only temperature and relative humidity (45), is therefore often used as a simpler alternative, including for heat advisories by the National Weather Service and public service campaigns by the U.S. Occupational Safety and Health Administration (OSHA) (46).

Similarly, ‘extreme’ heat can be defined in many ways: as relative or absolute; as daily minimum, mean, or maximum; and as single or multi-day events (47-49). Because of regional differences in sensitivity to heat, relative thresholds are preferable over absolute ones when defining extremes over large spatial scales (49), with the 95^th^-percentile level a commonly used measure (47,48). As we are primarily interested in workplace exposure, we used daytime statistics – the 95^th^-percentile of the MJJAS daily maximum HI and the frequency of 3-day heat waves above this level – as our measure of extreme heat. However, it is worth noting that nighttime conditions play an important role in modulating recovery rates (20,49), and are changing at different rates than daytime conditions (50).

### Historical heat exposure

To calculate past exposure to levels of extreme heat, we used 1979-2013 3-hourly surface air temperature and relative humidity data from the NCEP North American Regional Reanalysis (NARR) (51). The NARR data are available on a Lambert conformal grid, which we regridded to a 0.25°x0.25° rectilinear grid through bilinear interpolation. County-level values were calculated as the average of all grid points contained within a county, leading to a few missing values for very small counties. The Heat Index was calculated using the National Weather Service HI algorithm (52). We first calculated 3-hourly HI and then estimated daily mean, minimum, and maximum to preserve the co-variability between temperature and relative humidity.

### Future projections

Future climate projection data were obtained from 19 models^1^ in the Coupled Model Intercomparison Project 5 database (53) for the business-as-usual scenario Representative Concentration Pathway (RCP) 8.5. From this, we constructed a global warming temperature pattern (54) for each of the models by linearly regressing monthly-mean temperature against global annual mean temperature over the period 2006-2100. We then took the multi-model mean of these spatial patterns and scaled these to get the global warming pattern associated with a 2°C or 4°C global mean warming compared to late 20^th^-century. Under business-as-usual emissions (RCP8.5), 2 °C of global annual mean warming is projected to occur by 2055 (2042-2068), and 4 °C of warming by 2097 (2075-2132). Even in an emissions scenario aiming to stabilize greenhouse gas concentrations by mid-21^st^ century (RCP4.5), global mean temperature could rise by 2°C as early as 2052 (55). For relative humidity there is much larger inter-model spread in the spatial patterns of change (56), so instead we assumed a spatially uniform decrease in relative humidity of 1%/°C global warming (Supplementary Fig. 1). The conclusions of our study are robust across scenarios of relative humidity change ranging from −2%/°C to +2%/°C of global annual mean temperature change (Supplementary Fig. 7).

Because observed and projected changes in the diurnal temperature range are small compared to changes in daily average temperature (57,58), we assumed that future changes in temperature and relative humidity are distributed evenly throughout the day. We therefore added the change in the (2°C or 4°C warmer) temperature climatology and uniform relative humidity decrease to the observed (1979-2013) sub-daily NARR data, thus preserving the present-day variability on sub-daily to interannual time scales. From these 3-hourly data we again estimate daily mean, minimum, and maximum HI.

### Risk levels for heat exposure & adaptation measures

Guidance on human heat exposure in working populations, including from the World Health Organization and ACGIH (which is similar to NIOSH guidance), is based on maintaining the core body temperature within a safe range (e.g., within 1 °C of normal [37 °C] for unacclimatized individuals) (42,43,59). The recommended heat exposure levels, such as the ACGIH Threshold Limit Value (TLV) for heat stress – that is, the heat level to which nearly all heat-acclimatized, hydrated, and healthy workers can be exposed day after day without adverse health effects – are based on findings from human laboratory studies that examined the effect of exposure to different ambient temperature and humidity conditions under different physical activity and clothing scenarios on the ability to maintain the core body temperature within a safe range (43). We used an implementation of the ACGIH TLV intended for computing time-weighted average exposure levels and adapted for use with the HI assuming sun exposure (60), to compute heat stress TLVs under different scenarios.

For the baseline scenario, we assumed – based on the literature (61) – that workers are acclimatized and perform work activities in a 90% work/rest cycle at a moderate metabolic rate (300 Watt), spend breaks in the shade, and wear double-layer protective clothing. This resulted in a baseline TLV of 83.4 °F.

Short of stopping work altogether in places of high ambient HI, work practices can be modified in several ways that would lower heat stress and the risk for adverse health outcomes (62). With the understanding that these modifications may be costly or impractical, we considered the following options: slowing down the pace of work to a low metabolic demand; reducing work effort to a work/rest cycle of 50% (i.e. working half of the time); changing clothing ensembles to a more breathable single-layer garment; and taking breaks in an air-conditioned environment. The associated TLVs are shown in Table 1; plots of hourly work allowances for all the different scenarios are shown in Supplementary Fig. 2.

Next we calculated the number of days that daily mean MJJAS HI is above recommended TLV, for present-day and future climates, based on the baseline scenario and combinations of different adaptations. We used daily mean instead of daily maximum HI because worker exposure is spread out over multiple hours, and workplaces may already shift workers’ schedules to limit exposure during the hottest parts of the day (9,17).

## Results

### Agricultural workers in the United States

**Figure 1 –:**
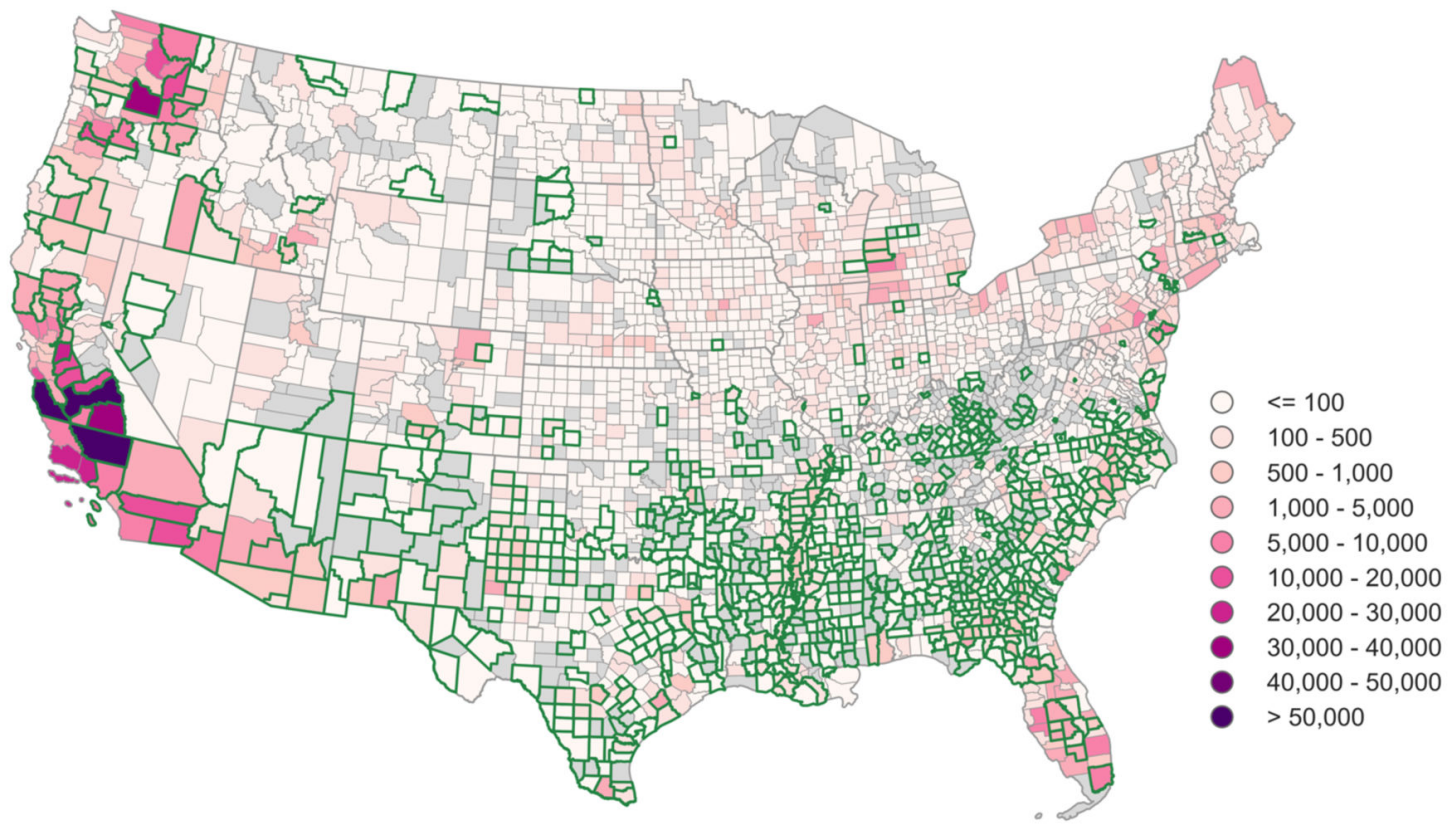
Spatial distribution of agricultural workers and social vulnerability. Number of summertime hired crop workers (MJJAS maximum of 2009-2018 average monthly values) as reported by the Bureau of Labor Statistics Quarterly Census of Employment and Wages (24). Counties outlined in dark green are in the upper quartile of the Center for Disease Control’s Social Vulnerability Index (41), indicating low community resilience to disaster events. Counties with no employment data are shown as missing values in gray.

As shown in Fig. 1, U.S. counties with the highest number of agricultural works are primarily along the West Coast (California, Oregon, Washington) and in Florida. Many of the counties with the highest levels of social vulnerability are also counties with high numbers of crop workers.

### Current and future heat extremes

**Figure 2 –:**
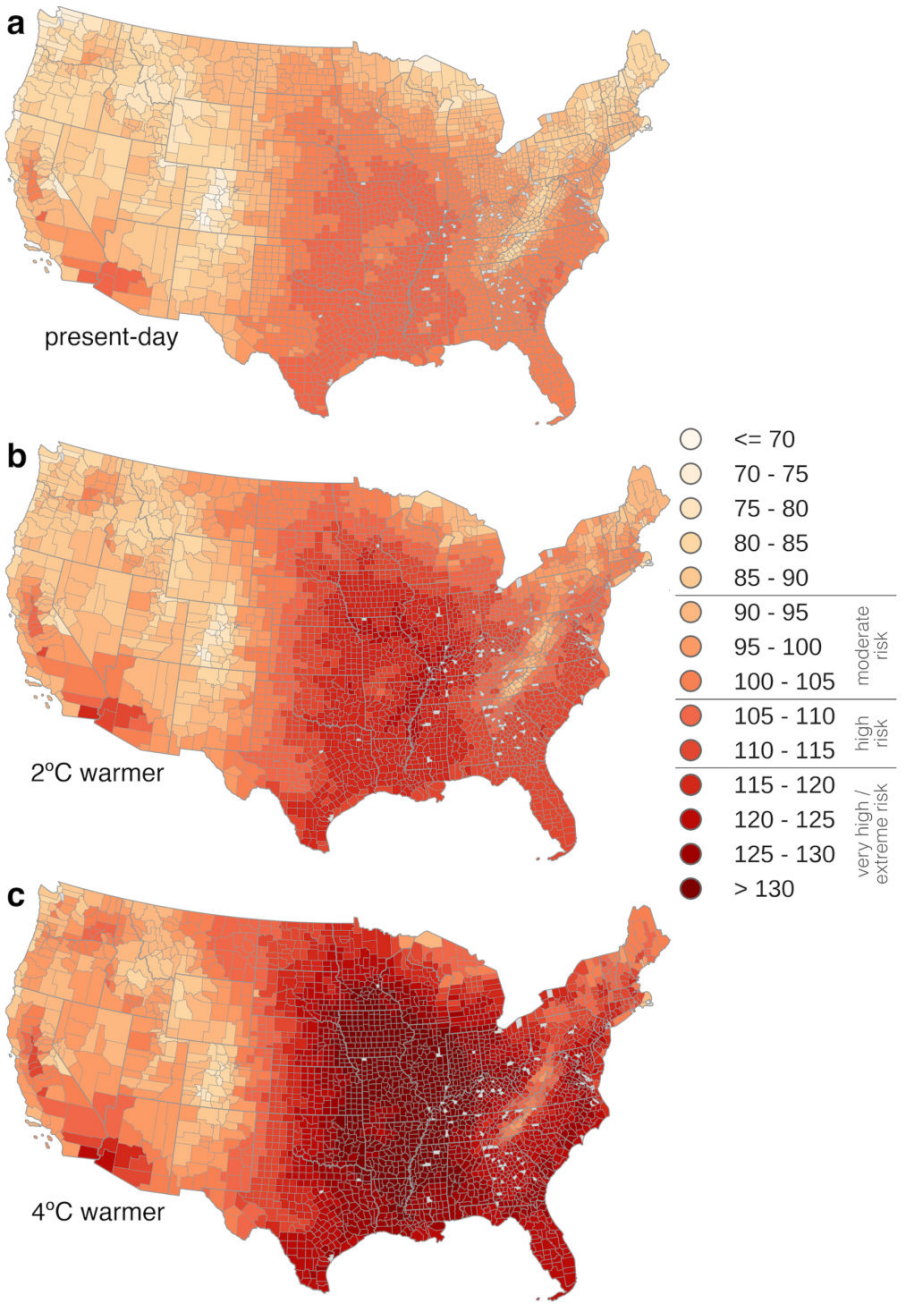
Present-day and projected Heat Index extremes. 95^th^-percentile of summertime (MJJAS) daily maximum Heat Index (°F) for **a** present-day observed (1979-2013), **b** projected with 2°C of global annual mean warming, and **c** projected with 4°C global annual mean warming (see Methods). Counties that contain no climate data grid centers are shown as missing values in gray. Color bar labels indicate the risk levels of the OSHA heat guidance for outdoor workers.

**Figure 3 –:**
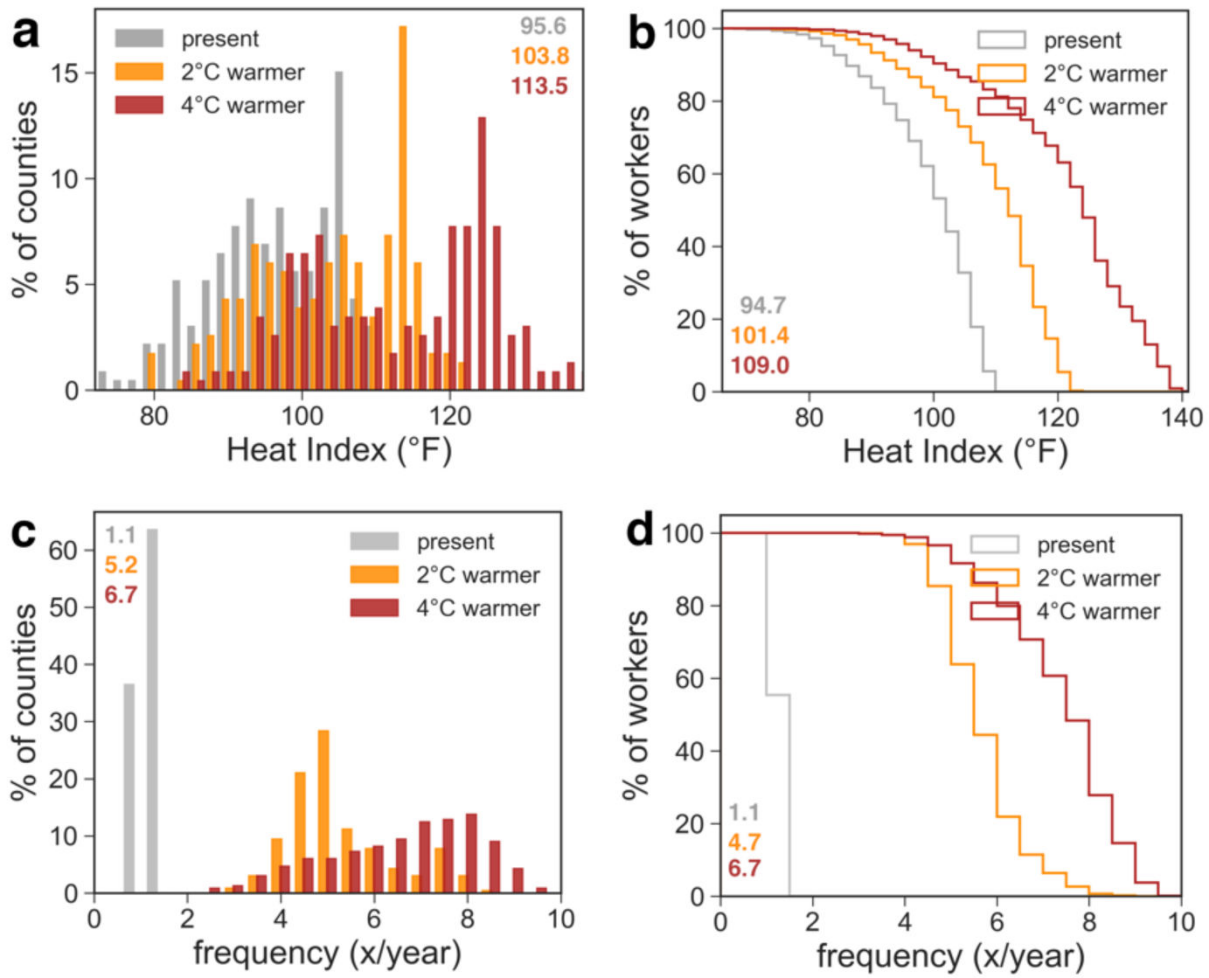
County and worker exposure to extreme heat levels and heat waves. **a,b** 95^th^-percentile of summertime (MJJAS) daily maximum Heat Index (°F) for present-day observed (gray), 2°C of global annual mean warming (orange), and 4°C of global annual mean warming (red) as **a** number of counties with ≥500 crop workers (%) and **b** cumulative number of workers (%); the percentage of **c** counties and **d** workers that experience a daily maximum HI for three or more days in a row that exceeds the *present-day* 95^th^-percentile level – colors and distributions the same as in **a,b**. The numbers in each corner indicate the county- (**a,c**) and worker- (**b,d**) weighted average.

Between 1979 and 2013, the average U.S. crop worker experienced summertime heat extremes of 94.7 °F HI – which OSHA considers to be of moderate risk – but the spatial variability in these extremes is high (Fig. 2a). Extreme heat is, not surprisingly, most severe in the South, southern Midwest, central California, and the coastal Southwest. Amongst the twenty counties with the most workers, heat extremes range from 78.1 to 109.2 °F (Table 2). Of all 233 counties with more than 500 crop workers, 24 have heat extremes above the OSHA ‘high risk’ level (105 °F; Fig. 3a).

With 2 °C of global annual mean warming, the levels of extreme summer heat will increase markedly (Fig. 2b). The average U.S. crop worker will face heat extremes of 101.4 °F HI. In the top twenty high-employment counties, the highest heat extremes are found in Imperial County, California, where they exceed the OSHA ‘very high/extreme’ risk level (115 °F); the only counties with heat extremes below the OSHA risk levels (<90 °F) will be located in Oregon and Washington (Table 2). Half of the 233 counties with ≥500 workers would have heat extremes at the OSHA ‘high risk’ level or above.

In a 4 °C warmer world, most of the continental U.S. east of the Rockies will have summertime levels of heat that are considered ‘very high/extreme’ by OSHA (Fig. 2c). The Mississippi Delta region in particular stands out for its high heat. It is worth noting that even though relatively few crop workers are located here, this area is one of general high vulnerability to extreme events (Fig. 1), making this a high-priority area for implementing heat resilience measures. Assuming current employment patterns, a majority of crop workers will experience ‘very high/extreme’ heat risk in the summertime growing season (Fig. 3). Of the twenty high-employment counties listed in Table 2, only one (Chelan, Washington) will have heat extremes that do not exceed OSHA risk levels.

Presently, in most counties, multi-day heat events only occur about once or twice a year (Fig. 3). With 2 °C of global warming, they will occur on average five times more often. With 4 °C of global warming, all counties will experience these types of events at least twice and up to ten times per year. Notably, in the Southeast of the U.S. the number of distinct heat events starts to decrease with higher degrees of warming, as longer and longer heat waves string together into single events (Supplementary Fig. 3).

### Days not fit for work

**Figure 4 –:**
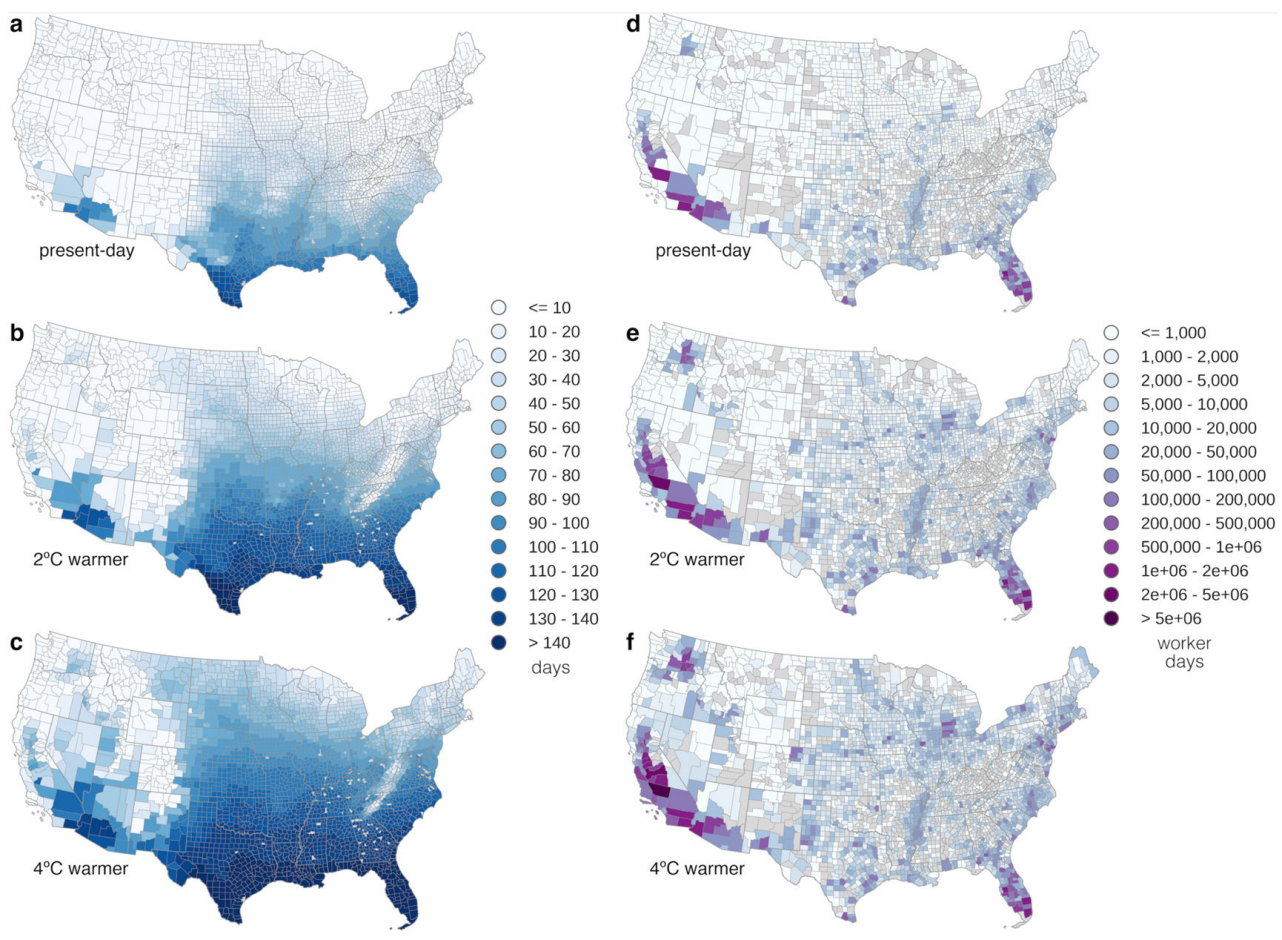
Present-day and projected worker exposure to unsafe heat levels. **a-c**, number of days each summer (MJJAS) that the daily mean Heat Index exceeds the baseline Threshold Limit Value (83.4°F; see Methods) for **a** present-day observed (1979-2013), **b** projected with 2°C of global annual mean warming, and **c** projected with 4°C global annual mean warming (see Methods); **d-f**, as **a-c** but showing number of worker days, based on present-day crop worker employment levels (Fig. 1). Note the nonlinear color scale for the number of worker days. Counties with no employment data or that contain no climate data grid centers are shown as missing values in gray.

**Figure 5 –:**
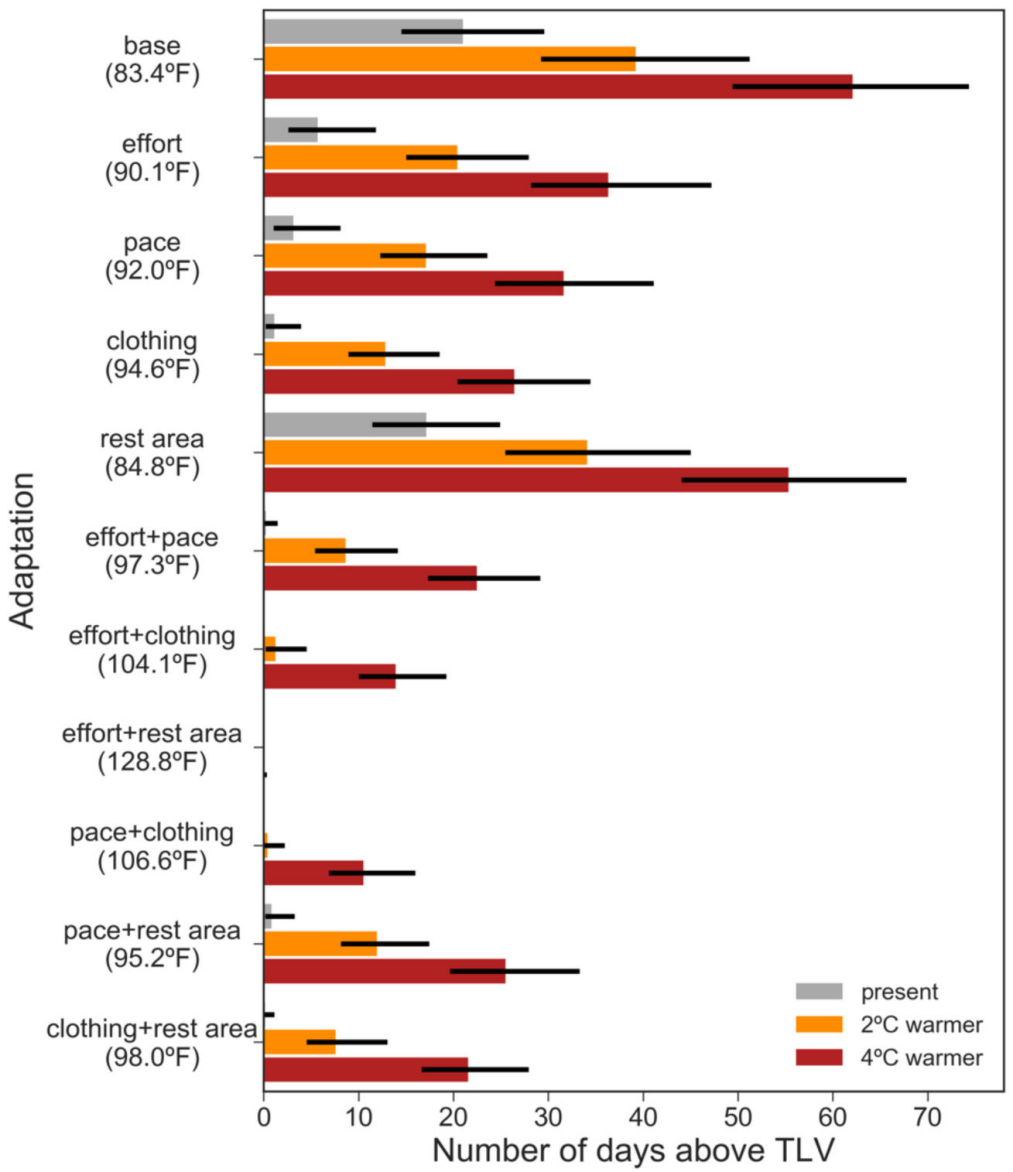
Effect of on-farm adaptation measures on reducing heat risk. Median number of days per summer (MJJAS) that the average U.S. crop worker is exposed to a daily mean Heat Index higher than the TLV for their physical activity and clothing levels (values in parentheses; see Methods), for present-day observed (gray), 2°C of global annual mean warming (orange), and 4°C of global annual mean warming (red). The error bars indicate the 5^th^ and 95^th^-percentile over the 35 years of observed and projected summers. The tested adaptation scenarios are: reduce effort (time worked/hour) from 90% to 50% (effort); reduce pace from moderate to light (pace); rest in AC instead of in the shade (rest area); wear single-layer instead of double-layer clothing (clothing); and combinations of these (see Table 1). When three or more adaptations are combined, worker exposure becomes (nearly) zero across all scenarios, so these are not plotted here.

The heat risk levels used by OSHA provide a first order estimate of when workers and employers should pay heed to worker safety, but recommendations based on these levels are generic, and fatalities have occurred at levels considered to be low risk by OSHA (<91° F) (63). To estimate heat risk in a more conservative and granular way, we calculated the number of days the mean HI is above the ACGIH TLV (see Methods). At present, primarily southern California and the Southeast have high numbers of days above the TLV (Fig. 4a,d). The average U.S. crop worker is exposed to 21 unsafe working days each summer growing season (out of 153 total) (Fig. 5). Of the twenty counties with the highest number of crop workers, Riverside CA has on average more than one month of unsafe working days, and both Imperial CA and Hillsborough FL have over three months of unsafe heat levels (Table 2).

In a 2 °C warmer world, more northern growing regions such as eastern Washington and New Jersey will also begin to see unsafe heat environments regularly, resulting in high numbers of exposure across the country (Fig. 4b,e). On average, crop workers will experience 39 days above safe heat levels (Fig. 5). Of the top twenty high-employment counties, four more (Kern, Merced and Stanislaus in California, and Benton in Washington) will have on average over a month of unsafe working days (Table 2).

With 4 °C of global annual mean warming, all high-employment counties will have at least one unsafe working day (Table 2). In the southernmost U.S., the daily mean HI will exceed the TLV on all days of the growing season (Fig. 4c,f). Assuming no spatial or seasonal modifications to cropping patterns, the average agricultural worker will labor 62 days in an unsafe thermal environment. High worker exposure in California, Arizona, Florida, and Washington in particular warrants attention (Fig. 5).

### Effect of adaptation measures

Work practices can be modified in several ways to lower heat stress and the risk for adverse health outcomes (62). Of the individual adaptive measures that we tested (Table 1), switching to more breathable clothing is most effective at reducing the exposure to unsafe heat levels, closely followed by reducing either pace or effort (Fig. 5). Switching to single-layer clothing more than halves the average worker exposure to 13 days (down from 39) in a 2 °C warmer world, and reduces it to 26 days (down from 62) in a 4 °C warmer world, though the Southeast in particular remains a hotspot for unsafe thermal environments (Supplementary Fig. 4-6).

When two adaptive measures are jointly implemented, the combination of resting more and resting in air-conditioning eliminates heat risk entirely (Fig. 5). Combining working less or at lower pace with wearing single-layer clothing significantly reduces heat risk. In these scenarios, even with 4 °C of global warming, heat exposure is lower than for present-day climate and working conditions (Fig. 5; Supplementary Fig. 6).

## Discussion

Discussions of the future of food in a changing climate (64) have largely ignored the important role of workers and their particular vulnerabilities. In this study, we combined climate projections with occupational health and safety guidelines to estimate the increase in health risks from heat exposure for U.S. agricultural workers with 2 and 4 °C of global warming. We find that by the middle of the century, half of agricultural counties will experience ‘high risk’ heat extremes (Figs. 2, 3), and multi-day heat events will occur five times more often. In the Southeast, where social vulnerability to disaster is high (Fig. 1), the entirety of the growing season will be considered unsafe for agricultural work with present-day work practices. Regions like the Northwest that are less accustomed to heat but that have high numbers of agricultural workers will start to see increasingly unsafe conditions as well (Fig. 4).

We tested the effectiveness of various workplace adaptation measures in offsetting this increased risk. The single most effective adaptation measure is for workers to switch to more breathable clothing, as this allows workers to generate less metabolic heat and more readily cool down (Fig. 5) (9,43). However, it is important to note that protective clothing and personal protective equipment (PPE) worn by crop workers often serves to shield against harmful agents, including dust, pesticides, nicotine, and UV radiation (18). Absent appropriate training and advances that make PPE both breathable and a barrier to chemicals, workers may face a trade-off between safety from heat or from chemicals. When combining adaptive measures, we found that, as expected, slowing the pace of work and reducing work effort, are effective at reducing exposure to unsafe heat levels. Barring changes in cropping patterns and work practices (see below), these measures could significantly hurt farm productivity, farm worker earnings, and/or labor costs for the employer.

### Limitations and future research

There are several shortcomings to our approach that likely lead to an underestimate of growing health risk. Our use of daily mean HI and assumption that workers are acclimatized to their environment ignore the impact of sub-daily and sub-seasonal (48) heat extremes. Our approach also does not include the compounding effects of nighttime heat and multi-day heat events (20,48). Furthermore, we used one level to distinguish safe from unsafe working days, but in reality health risk will continue to increase as the HI rises above the TLV. Finally, though the ACGIH framework is based on data from younger adults and assumes that workers are healthy and hydrated, in actuality individual, workplace, and community factors contribute to vulnerability (5,9,18,19,25,41) (Fig. 1). Each of these assumptions renders our results to be conservative estimates of the increase in heat exposure due to warming.

In addition, though it seems reasonable to assume that the QCEW data (Fig. 1) are broadly representative of the spatial distribution of agricultural labor in the U.S., some evidence suggests that workers not included in this dataset may be disproportionally located in certain geographies. For example, while California and Washington have the highest number of agricultural workers in the QCEW data (24), Georgia and Florida have in recent years had the highest number of H-2A visa workers (65). Further, the National Agricultural Workers Survey estimates the share of unauthorized workers to range from 28 to 57% across six U.S. regions (66). We are therefore unable to identify areas and populations beyond those included in this study for which preventative and protective measures should be prioritized (40).

We assumed in our analyses that spatial and seasonal cropping patterns will remain stationary. This is unlikely given climate (64,67), societal (68), and technological (69) trends, but to our knowledge no projections of future U.S. cropping patterns exist. Crops will be impacted directly by a changing climate (67), and it is likely that where, when, and what crops are planted will adjust in response (70,71). However, most studies on the impact of climate change on crops have focused on (labor-extensive) staple grains (64), such that the future of fruit, vegetable, legume, and nut production (which already falls short of dietary needs (72)) in a vastly different climate remains mostly unexplored. Predicting shifts in cropping patterns is further complicated by the interconnectedness of local and global labor and commodity markets (68,73). Labor-saving and laborenhancing innovations, including mechanization and robotics, could also drastically alter (or eliminate) farm worker practices (69,74), including human work pace and effort.

While the direct effects of heat on agricultural worker health is of primary importance, downstream and modifying effects must also be acknowledged. For example, it is not clear how the health effects of rising heat extremes will interact with other climate change threats to occupational health (75), such as more frequent wild fires in the western U.S. (76), worsening air quality (77), higher asthma rates from pollen and dust (78), growing pest pressure (79), and increased pollutant toxicity with heat (80). More research is needed on how trends in cropping patterns, technology, markets, and other climate impacts will interact. This requires transdisciplinary collaboration between multiple sectors, including public health, climate science, agronomy, economics, and farmworkers and the agricultural industry and should explicitly include consideration of impacts on worker health and well-being.

### Recommendations

Approaches at multiple levels are needed to reduce agricultural worker health risks from heat stress now and in the future. A standard approach to framing occupational health and safety interventions is the industrial hygiene hierarchy of controls, in which stronger controls (e.g. reduction in heat exposure and engineering controls) are those that rely less on individual behavior change than weaker controls (e.g. PPE use) (11).

In the near term, ‘weak’ controls at the individual and workplace level, such as improved PPE use, rest practices and work hours, could effectively reduce heat exposure (81). At the community level, significant gains can be made by improving farmworker housing conditions, which influence rest and recovery and can offset the negative impacts of nighttime heat (20). However, further work is needed to advance these heat stress controls. For example, advances in engineering and materials science are needed to develop and optimize PPE that is both breathable and appropriately protective for various hazards (including pesticides). More work needs to be done to characterize the factors that influence the relationship between indoor and outdoor heat exposure in rural settings, such as safety concerns for opening windows and the limited effectiveness of small window unit air conditioners (20).

Incentivizing changes in heat stress controls is difficult without a strong regulatory framework. Policies at the workplace, state, and federal level are needed that address differences in risk and vulnerabilities in different settings. Only two states – California and Washington – have outdoor occupational heat standards in place (9), yet our results clearly show that agricultural workers will soon be at significant risk across the entire country. NIOSH, scientists, and civic advocacy groups have repeatedly petitioned the U.S. government to implement outdoor occupational heat standards that would require, amongst others, heat-appropriate breaks, appropriate PPE, shade, and hydration; worker training and hazard notification; early warning systems; and medical and exposure monitoring (42,82). Though policies exist in some states that require temporary farmworker housing to be maintained within a certain temperature range (83), more research is needed to support further lowering these heat exposure limits.

In the long term, controls that rely on individual behavioral change will no longer be sufficient to protect workers. With 2 to 4 °C of global warming, large parts of the country will experience the kind of conditions that currently result in temporary work bans in countries like China, India, Saudi Arabia, and the United Arab Emirates (84). Though a certain degree of warming cannot be avoided, extreme impacts on crop worker health, and agriculture more broadly, can ultimately only be reduced through strong climate change mitigation, i.e., rapid reduction of carbon emissions and increased carbon sequestration (11,85). In addition, disparities that ultimately impact the safety and health of agricultural workers cannot be full addressed without also addressing the social, economic, and political context (21,22). Climate mitigation therefore needs to be paired with systemic change around drivers of (climate) vulnerability – including poverty, immigration policy, and health care inequalities – on top of the regulatory and adaptive measures outlined above.

## Conclusion

Climate change at the current pace will double crop worker heat risk by the middle of this century and triple it by the end of it. Our results demonstrate that adaptation at the worker and workplace level can mitigate this risk but only through an extensive restructuring of agricultural labor. To safeguard the health and well-being of millions, the full spectrum of risk-reduction levers therefore needs to be employed, including policies promoting the social, economic, and political empowerment of vulnerable populations and rapid action on climate change. In the near term, building inclusive transdisciplinary collaborations that include farm workers at the table will help ensure their voices are incorporated in discussions of growing food in a changing climate.

## Supplementary Material

Supplementary Material

## Figures and Tables

**Table 1 –:** On-farm worker adaptations to reduce heat risk. Various modifications to worker behavior that can be implemented to reduce crop worker heat risk and their associated ACGIH Threshold Limit Value.

Adaptation scenario	Assumptions	TLV (HI in °F)
Baseline	Work 90% effort, at moderate pace, wearing double-layer clothing, resting in shade	83.4
Effort	Work 50% effort, at moderate pace, wearing double-layer clothing, resting in shade	90.1
Pace	Work 90% effort, at light pace, wearing double-layer clothing, resting in shade	92.0
Clothing	Work 90% effort, at moderate pace, wearing single-layer clothing, resting in shade	94.6
Rest Area	Work 90% effort, at moderate pace, wearing double-layer clothing, resting in AC	84.8
Effort + Pace	Work 50% effort, at light pace, wearing double-layer clothing, resting in shade	97.3
Effort + Clothing	Work 50% effort, at moderate pace, wearing single-layer clothing, resting in shade	104.1
Effort + Rest Area	Work 50% effort, at moderate pace, wearing double-layer clothing, resting in AC	128.8
Pace + Clothing	Work 90% effort, at light pace, wearing single-layer clothing, resting in shade	106.6
Pace + Rest Area	Work 90% effort, at light pace, wearing double-layer clothing, resting in AC	95.2
Clothing + Rest Area	Work 90% effort, at moderate pace, wearing single-layer clothing, resting in AC	98.0

**Table 2 –:** Heat exposure levels in counties with most crop workers. Present-day observed and projected future 95^th^-percentile of summertime (MJJAS) daily maximum Heat Index (°F) and number of days above baseline TLV (83.4 °F; see Methods) in the twenty counties with the highest number of crop workers (Fig. 1). Colors indicate the OSHA risk levels of moderate (yellow), high (orange), and very high/extreme (red).

county	# of workers	95^th^-percentile MJJAS HI_max_			days/year above TLV		
		present	+2°C	+4°C	present	+2°C	+4°C
**Kern, California**	65492	98.5	102.5	106.3	24	55	90
**Monterey, California**	64796	89.9	93.7	97.6	0	2	14
**Fresno, California**	54804	93.6	97.8	101.9	2	13	45
**Tulare, California**	37956	91.4	95.5	99.5	0	8	37
**Yakima, Washington**	37761	83.8	89.4	95.2	0	1	8
**Ventura, California**	29196	90.3	94.4	98.5	0	3	17
**Santa Barbara, California**	23304	86.5	90.0	94.0	0	0	5
**San Joaquin, California**	21399	101.7	106.2	110.3	6	23	59
**Riverside, California**	15707	100.1	105.2	110.6	42	77	105
**Chelan, Washington**	14849	78.1	83.2	89.0	0	0	1
**Merced, California**	13011	103.1	107.2	110.9	20	54	90
**Stanislaus, California**	12580	101.7	106.1	110.1	15	45	80
**Santa Cruz, California**	12538	89.2	93.3	97.6	0	0	3
**Grant, Washington**	12398	92.1	98.2	104.4	4	20	48
**Marion, Oregon**	12092	82.7	89.0	96.9	0	0	4
**Madera, California**	12058	93.5	98.2	102.5	2	11	43
**Imperial, California**	11505	109.2	115.3	122.0	105	124	136
**Okanogan, Washington**	9761	79.3	84.8	90.9	0	0	3
**Hillsborough, Florida**	9644	103.6	112.7	123.1	113	137	148
**Benton, Washington**	8867	95.4	101.5	107.3	9	31	61

## Footnotes

1 ACCESS1-0, ACCESS1-3, CanESM2, CNRM-CM5, CSIRO-Mk3-6-0, GFDL-CM3, GFDL-ESM2G, GFDL-ESM2M, GISS-E2-H, GISS-E2-R, HadGEM2-AO, inmcm4, IPSL-CM5A-MR, IPSL-CM5B-LR, MIROC5, MIROC-ESM, MRI-CGCM3, MRI-ESM1, NorESM1-M
