# Supplementary material for: Work Adaptations Insufficient to Address Growing Heat Risk for U.S. Agricultural Workers
Source: Environ Res Lett. Author manuscript; Available in PMC 2020 Oct 29. (PMC7594196; doi:10.1088/1748-9326/ab86f4)
Supplement: Supplementary Material [file NIHMS1582713-supplement-Supplementary_Material.docx]

# Work Adaptations Insufficient to Address Growing Heat Risk for U.S. Agricultural Workers

Michelle Tigchelaar^1,2,*^, David S. Battisti^1^, June T. Spector^3,4^

^1^ Department of Atmospheric Sciences, University of Washington, Seattle, WA

^2^ now at: Center for Ocean Solutions, Stanford University, Palo Alto, CA

^3^ Department of Environmental and Occupational Health Sciences, University of Washington, Seattle, WA

^4^ Department of Medicine, University of Washington, Seattle, WA

**Supplementary Figures**


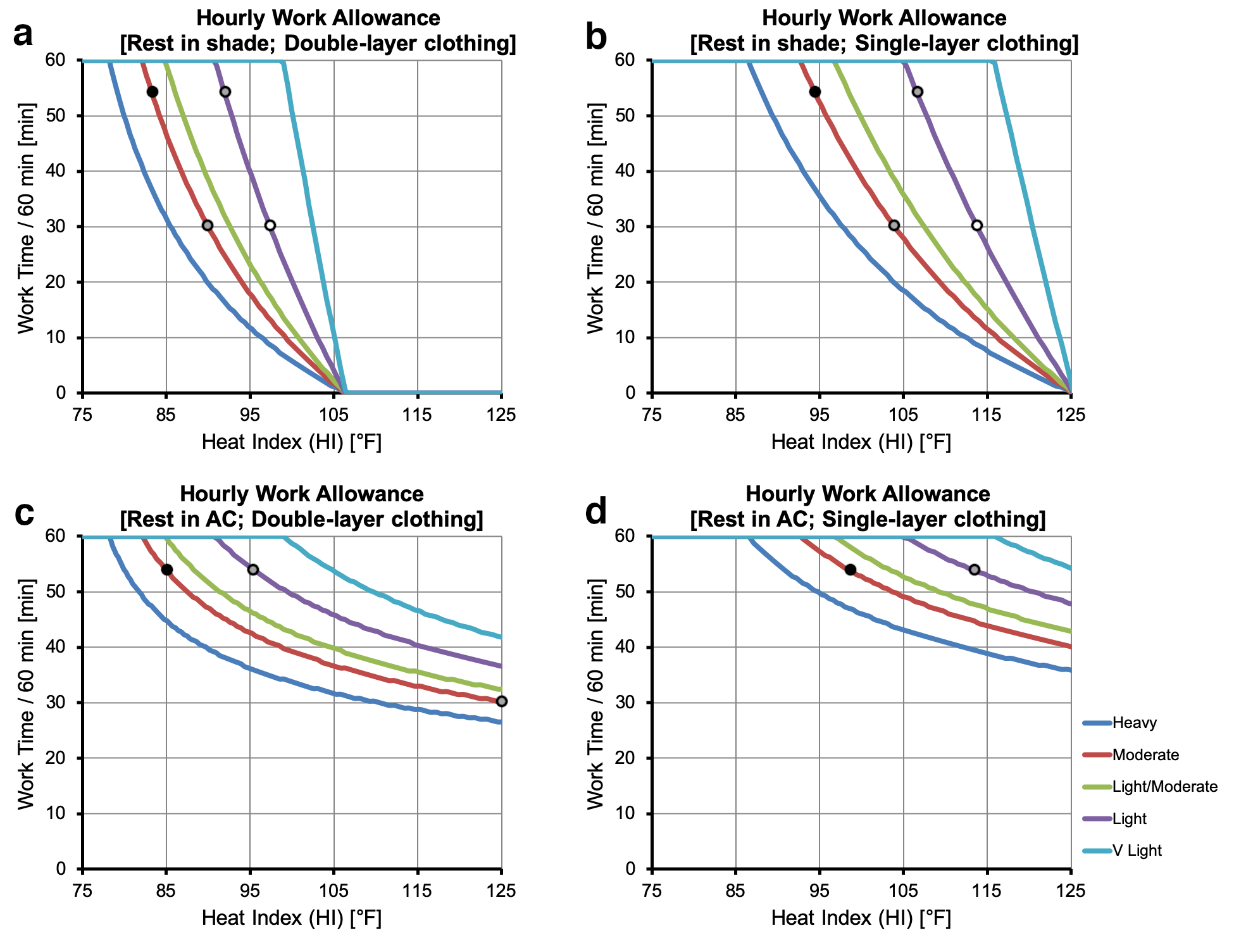


**Supplementary Fig. 1 – Hourly work allowance for different environmental, work pace, and clothing conditions.** Number of minutes per hour a worker can safely work at a given Heat Index (ºF) when working at very light (light blue), light (purple), light/moderate (green), moderate (red), or heaver (dark blue) pace. Values are calculated assuming **a** resting in shade, wearing double-layer clothing, **b** resting in shade, wearing single-layer clothing, **c** resting in AC, wearing double-layer clothing, and **d** resting in AC, wearing single-layer clothing. In each plot, the black dot indicates working at 90% effort (54min/hour) and moderate pace; gray dots indicate either reducing pace to light or reducing effort to 50% (30min/hour), and the white dot indicates doing both.


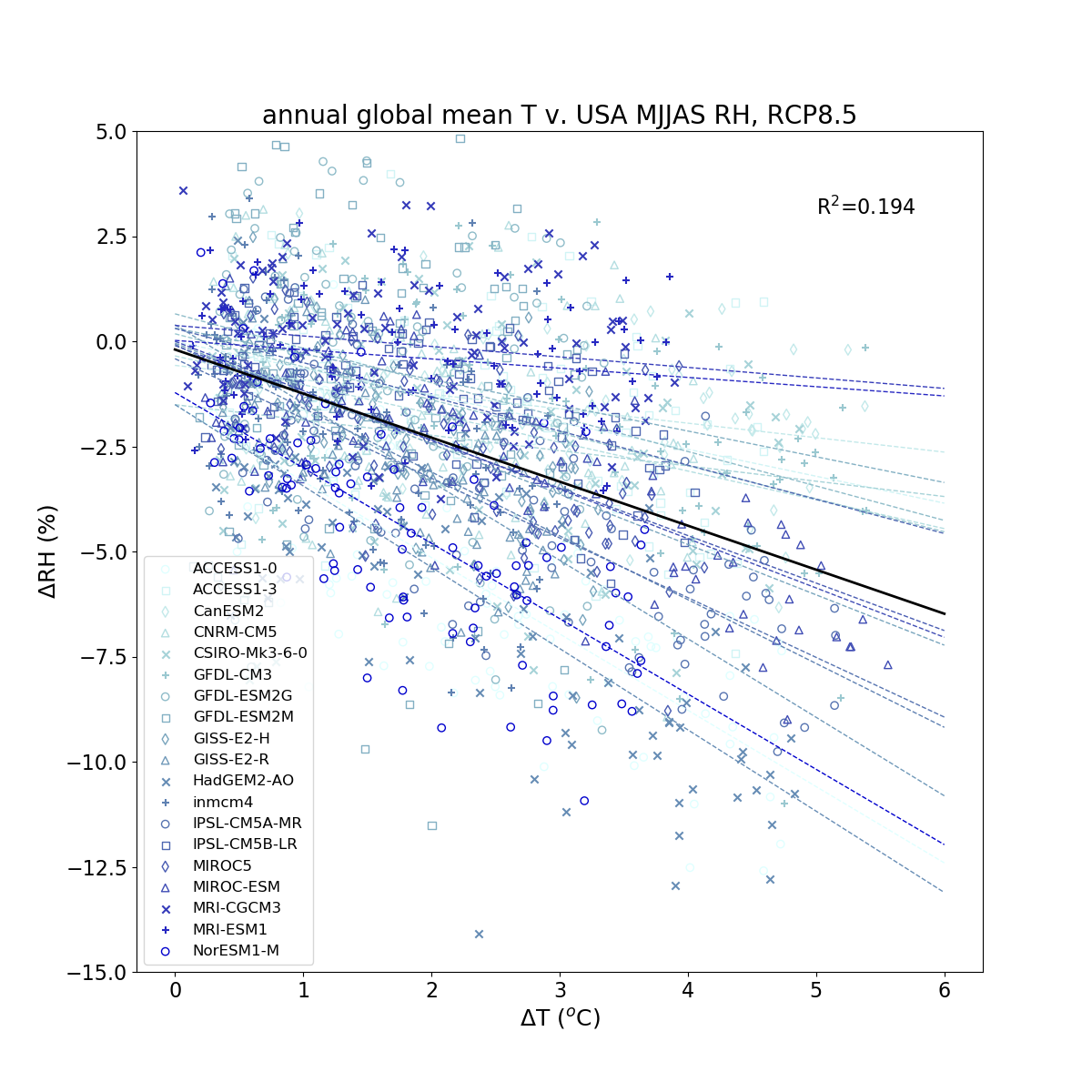


**Supplementary Fig. 2 – Change in U.S.-average relative humidity for a given amount of global warming.** Projected change in CONUS-average summertime (MJJAS) relative humidity for a given amount of annual mean global mean warming in each of the CMIP5 models included in our study, and for each of the projected years from 2006-2100. Colored lines show the linear fit through each model’s data. The solid black line indicates the multi-model mean, with associated R^2^ in the upper right corner. Based on the multi-model mean, we use a uniform -1% relative humidity change for each degree of global annual mean warming. The results are fairly insensitive to this value: results with -2%, 0%, +1% and +2% are shown for comparison in Supplementary Fig. 7.


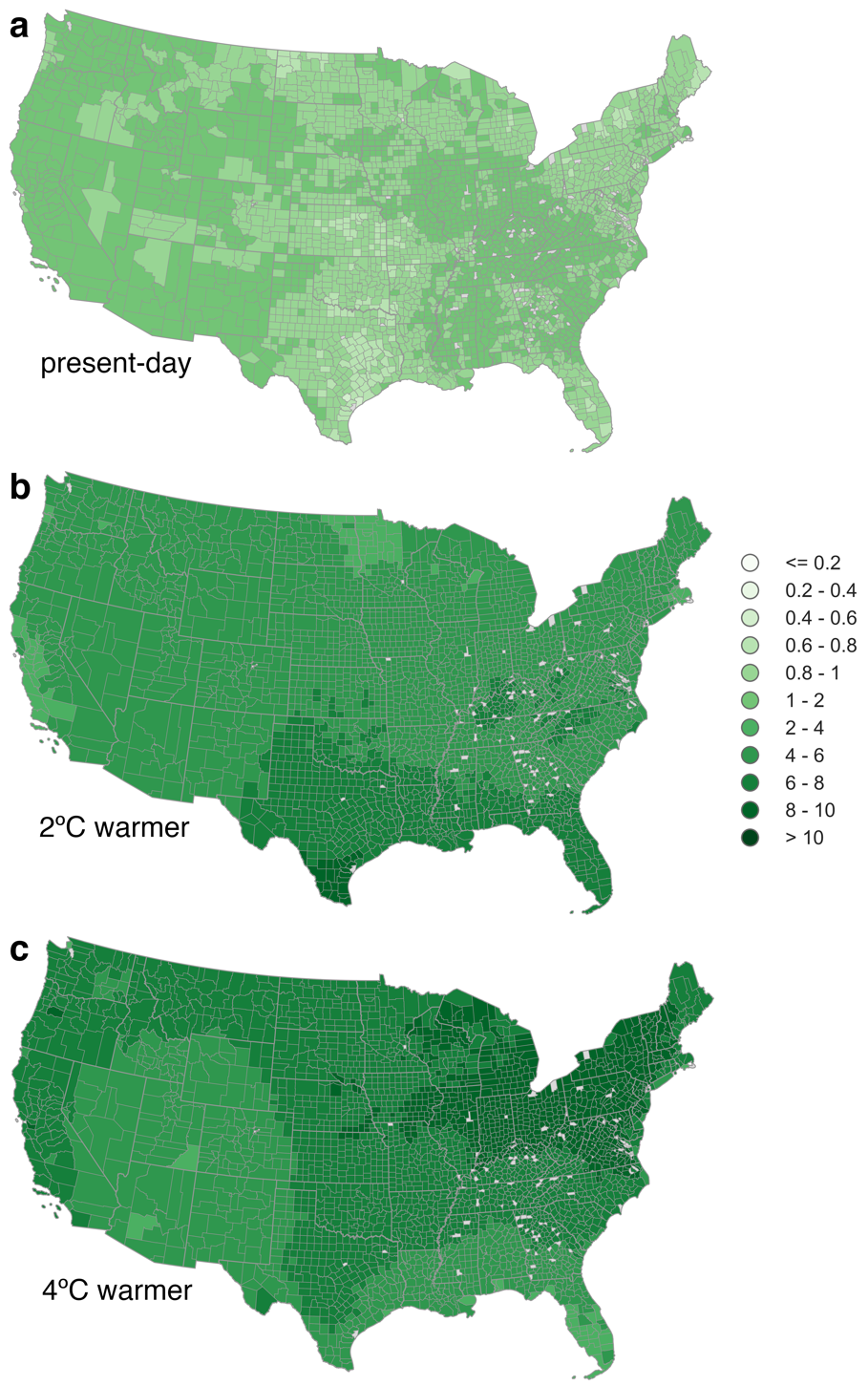


**Supplementary Fig. 3 ­– Present-day and projected frequency of heat waves of 3 or more days.** Average times per summer (MJJAS) that the present-day 95^th^-percentile of daily maximum Heat Index is exceeded 3 or more days in a row, for **a** present-day observed (1979-2013), **b** projected with 2ºC of global annual mean warming, and **c** projected with 4ºC global annual mean warming (see Methods). Counties that contain no climate data grid centers are shown as missing values in gray. At some locations the projected frequency decreases with higher degrees of global warming as heat waves string together into single events.


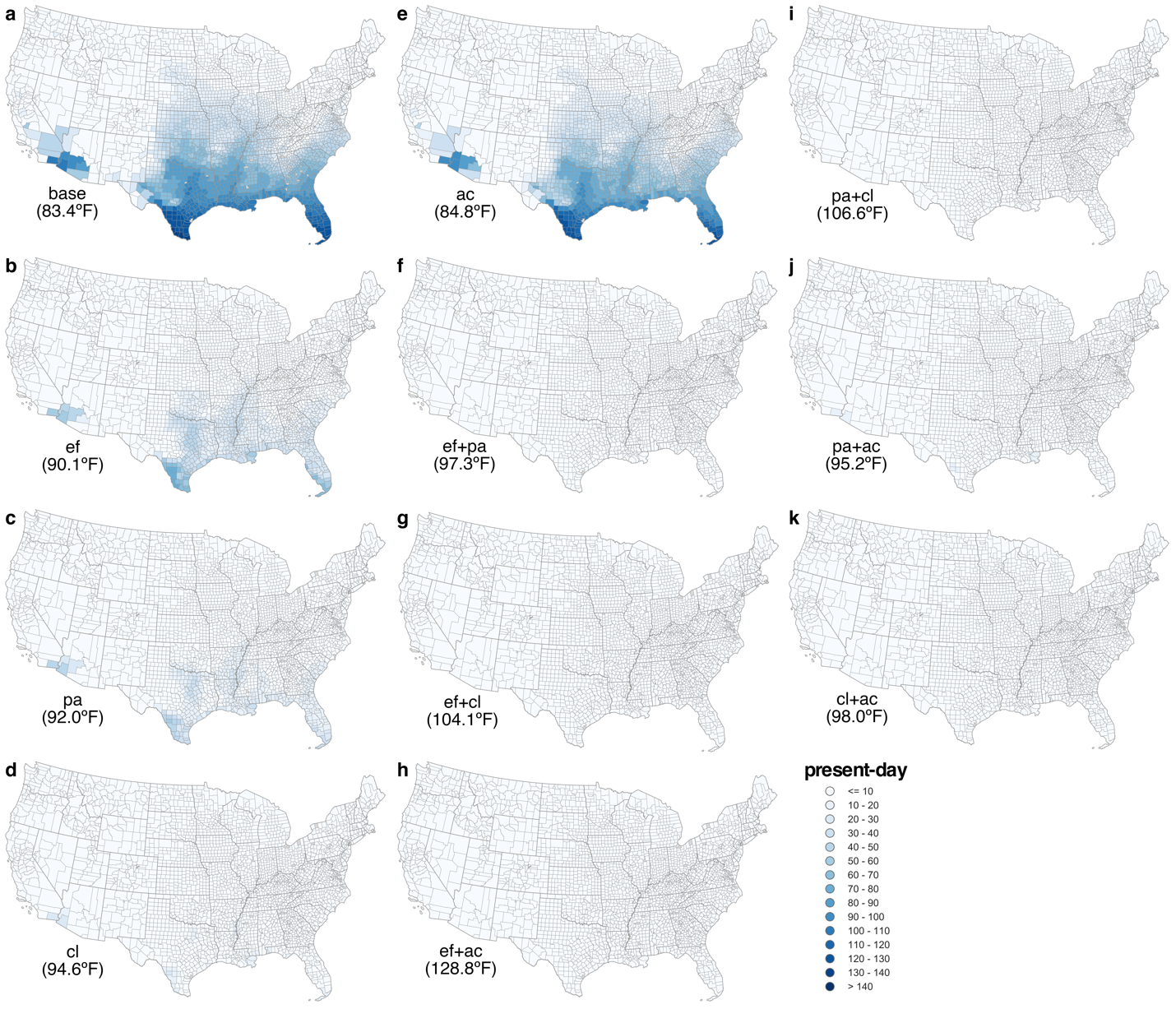


**Supplementary Fig. 4  – Present-day worker exposure to unsafe heat levels using various worker adaptations.** Number of days each summer (MJJAS) that the daily mean Heat Index in present-day observed climate (1979-2013) exceeds the Threshold Limit Value of **a** baseline conditions, **b** reduced effort, **c** reduced pace, **d** single-layer clothing, **e** resting in AC, **f** reduced effort and pace, **g** reduced effort and single-layer clothing, **h** reduced effort and resting in AC, **i** reduced pace and single-layer clothing, **j** reduced pace and resting in AC, **k** single-layer clothing and resting in AC (see Methods). Counties that contain no climate data grid centers are shown as missing values in gray.


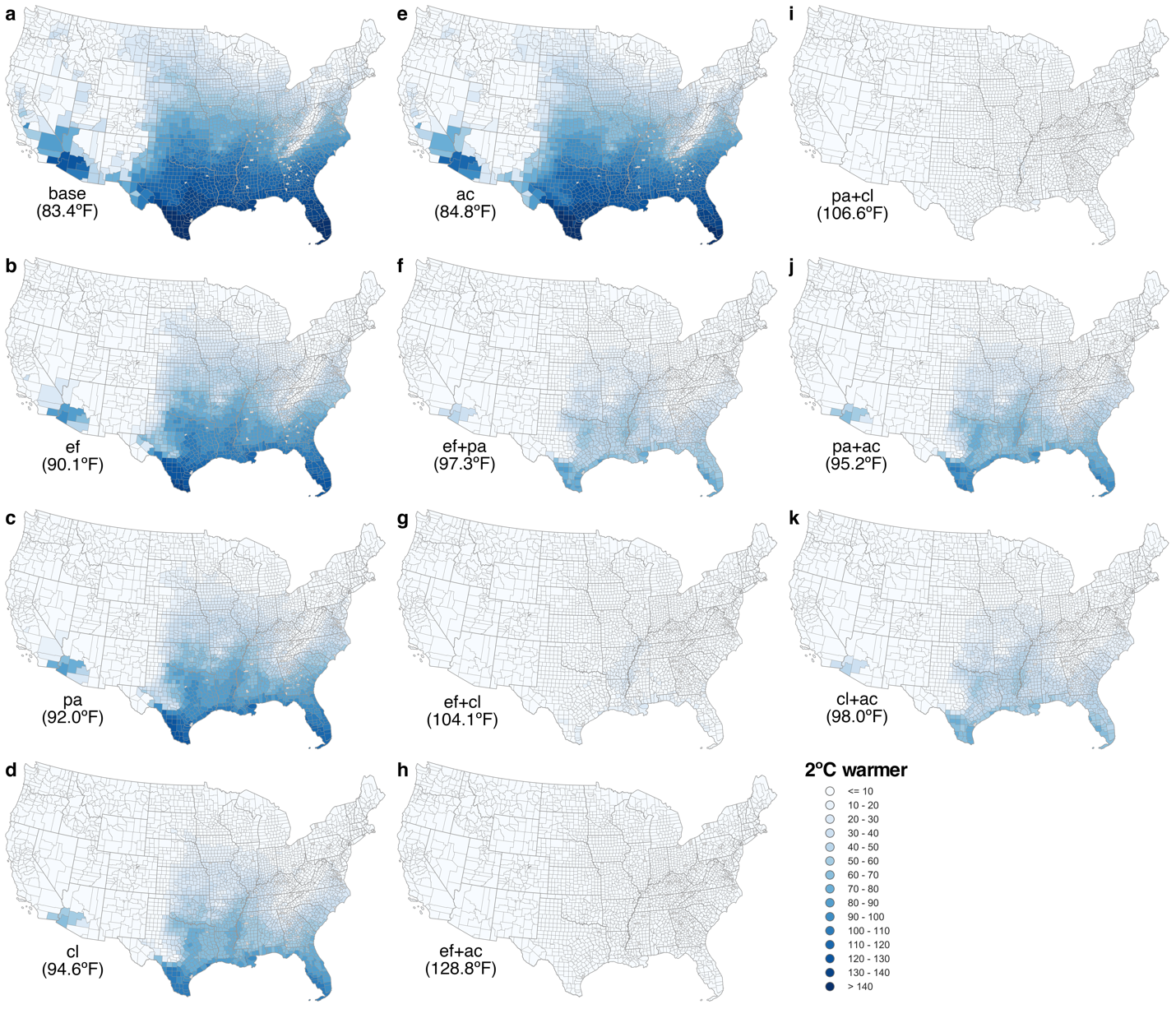


**Supplementary Fig. 5  – Worker exposure to unsafe heat levels using various worker adaptations with 2ºC of global warming.** Number of days each summer (MJJAS) that the daily mean Heat Index in a 2ºC warmer climate exceeds the Threshold Limit Value of **a** baseline conditions, **b** reduced effort, **c** reduced pace, **d** single-layer clothing, **e** resting in AC, **f** reduced effort and pace, **g** reduced effort and single-layer clothing, **h** reduced effort and resting in AC, **i** reduced pace and single-layer clothing, **j** reduced pace and resting in AC, **k** single-layer clothing and resting in AC (see Methods). Counties that contain no climate data grid centers are shown as missing values in gray.


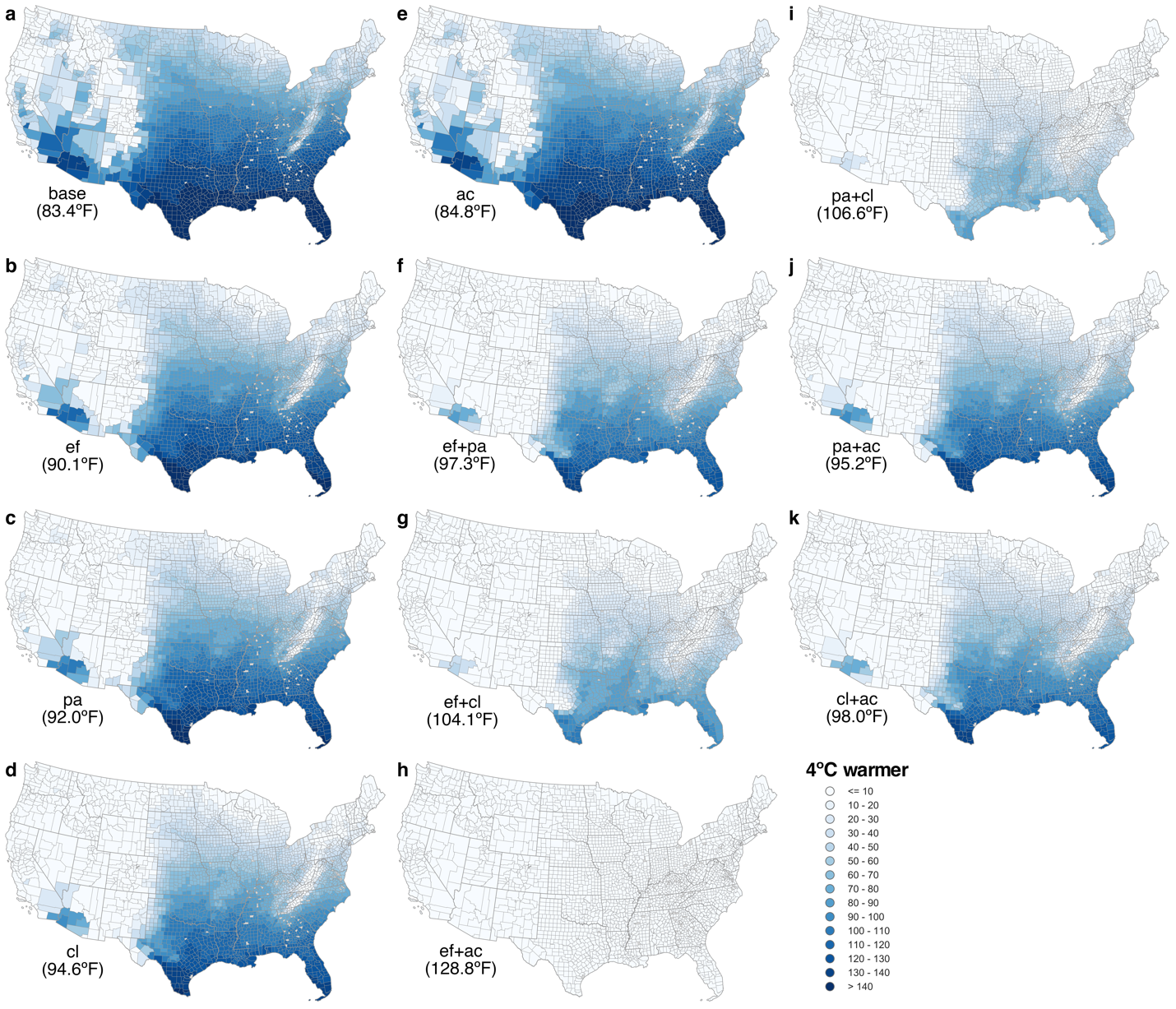


**Supplementary Fig. 6  – Worker exposure to unsafe heat levels using various worker adaptations with 4ºC of global warming.** Number of days each summer (MJJAS) that the daily mean Heat Index in a 4ºC warmer climate exceeds the Threshold Limit Value of **a** baseline conditions, **b** reduced effort, **c** reduced pace, **d** single-layer clothing, **e** resting in AC, **f** reduced effort and pace, **g** reduced effort and single-layer clothing, **h** reduced effort and resting in AC, **i** reduced pace and single-layer clothing, **j** reduced pace and resting in AC, **k** single-layer clothing and resting in AC (see Methods). Counties that contain no climate data grid centers are shown as missing values in gray.

**
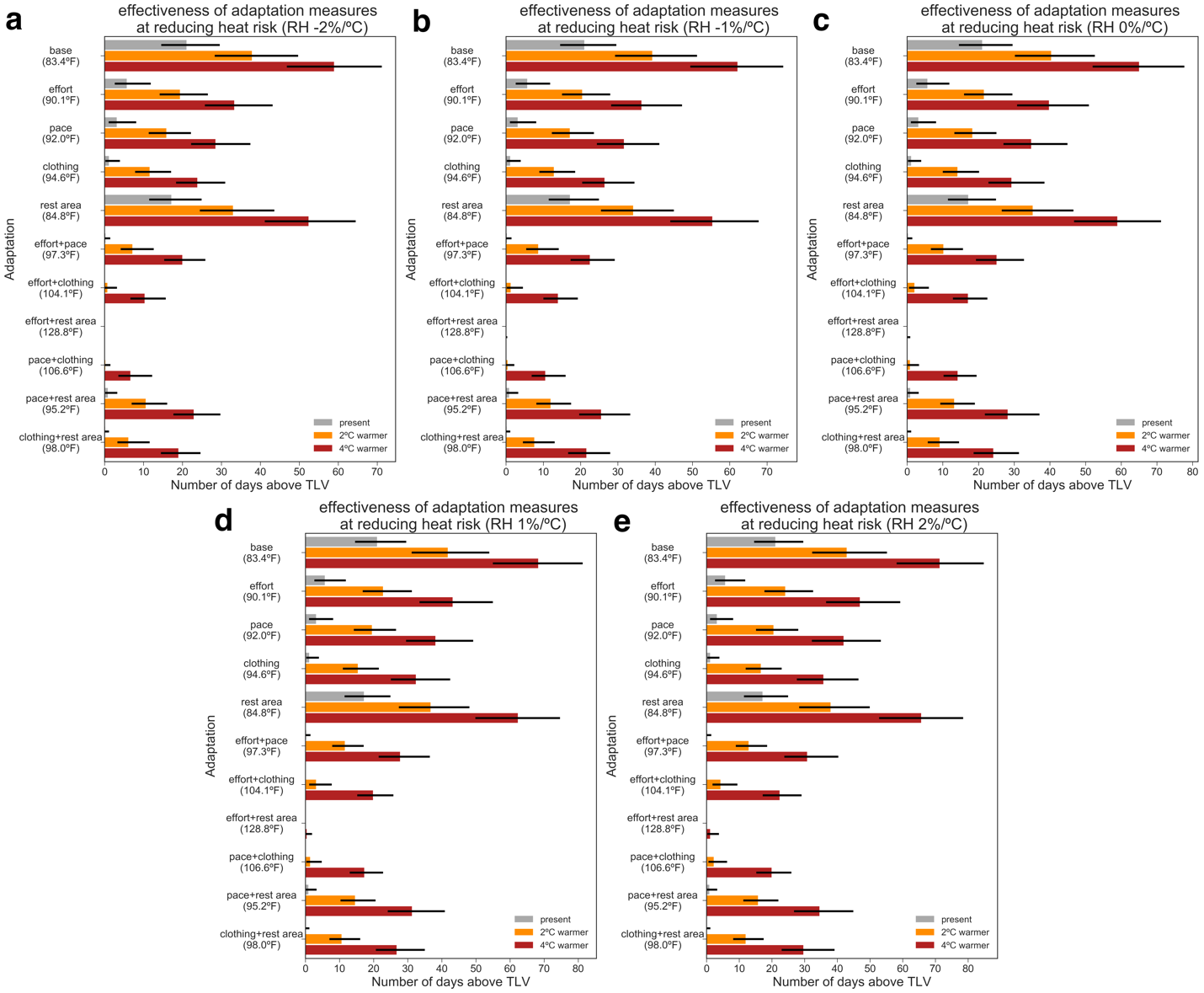
**

**Supplementary Fig. 7 – Effectiveness of on-farm adaptation measures at reducing heat risk under various scenarios of Relative Humidity change.** Median number of days per summer (MJJAS) that the average U.S. crop worker is exposed to a daily mean Heat Index higher than the TLV for their physical activity and clothing levels (see Methods), for present-day observed (gray)**,** 2ºC of global annual mean warming (orange), and 4ºC of global annual mean warming (red). The error bars indicate the 5^th^ and 95^th^-percentile over the 35 years of observed and projected summers. The tested adaptation scenarios are: reduce effort (time worked/hour) from 90% to 50% (effort); reduce pace from moderate to light (pace); rest in AC instead of in the shade (rest area); wear single-layer instead of double-layer clothing (clothing); and combinations of these. For each warming scenario, the relative humidity is uniformly changed by **a** -2%/ºC, **b** -1%/ºC (main paper), **c** 0%/ºC, **d** +1%/ºC, **e** +2%/ºC for each degree of global annual mean warming.
